# The *q*-Laguerre matrix polynomials

**DOI:** 10.1186/s40064-016-2178-5

**Published:** 2016-04-30

**Authors:** Ahmed Salem

**Affiliations:** Department of Basic Science, Faculty of Information Systems and Computer Science, October 6 University, Sixth of October City, Egypt

**Keywords:** *q*-Laguerre matrix polynomials, *q*-Gamma matrix function, Matrix functional calculus, Three terms recurrence relation, Rodrigues-type formula, 33D05, 33D45, 15A16

## Abstract

The Laguerre polynomials have been extended to Laguerre matrix polynomials by means of studying certain second-order matrix differential equation. In this paper, certain second-order matrix *q*-difference equation is investigated and solved. Its solution gives a generalized of the *q*-Laguerre polynomials in matrix variable. Four generating functions of this matrix polynomials are investigated. Two slightly different explicit forms are introduced. Three-term recurrence relation, Rodrigues-type formula and the *q*-orthogonality property are given.

## Background

The study of functions of matrices is a very popular topic in the Matrix Analysis literature. Some basic references are Gantmacher (1998), Higham (2008) and Horn and Johnson (1991). The subject of the orthogonal polynomials cuts across a large piece of mathematics and its applications. Matrix orthogonality on the real line has been sporadically studied during the last half century since Krein devoted some papers to the subject in 1949. In the last two decades this study has been made more systematic with the consequence that many basic results of scalar orthogonality have been extended to the matrix case. The most recent of these results is the discovery of important examples of orthogonal matrix polynomials: many families of orthogonal matrix polynomials have been found that (as the classical families of Hermite, Laguerre and Jacobi in the scalar case) satisfy second order differential equations with coefficients independent of *n* (Duran and Grunbaum 2005).

The second-order matrix differential equations of the form

1 $$xY^{\prime \prime }(x)+(A+I-\lambda xI)Y^{\prime }(x)+\lambda nY(x)=0,\quad n\in {\mathbb{N}}_0,\;A,Y(x)\in {\mathbb{C}}^{r\times r}$$

have been introduced and investigated in Jódar et al. (1994), where ${\mathbb{C}}^{r\times r}$ denotes the vector space containing all square matrices with *r* rows and *r* columns with entries in the complex number ${\mathbb{C}}$, $\lambda \in {\mathbb{C}}$ and *x* is a real number. The explicit expression for the *n*th Laguerre matrix polynomial $L^{(A,\lambda )}_n(x)$ which is a solution of the second order matrix differential equation (1), has the form

2 $$L^{(A,\lambda )}_n(x)=\sum _{k=0}^n{(-1)^k\lambda ^k\over k!(n-k)!}(A+I)_n(A+I)_k^{-1}x^k,\quad {\mathfrak{R}}(\lambda )>0$$

where $(A+I)_n=(A+I)(A+2I)\ldots (A+(n-1)I), n\in {\mathbb{N}}$ and $(A+I)_0=I.$ An explicit expression for the Laguerre matrix polynomials, a three-term matrix recurrence relation, a Rodrigues formula and orthogonality properties are given in Jódar et al. (1994). The Laguerre matrix polynomials satisfy functional relations and properties which have been studied in Jódar and Sastre (2001, 2004), Sastre and Jódar (2006a, b), Sastre and Defez (2006), Sastre et al. (2006).

Recently, *q*-calculus has served as a bridge between mathematics and physics. Therefore, there is a significant increase of activity in the area of the *q*-calculus due to its applications in mathematics, statistics and physics. The one of the most important concepts in *q*-calculus is the Jackson *q*-derivative operator defined as

3 $$(D_qf)(x)={f(x)-f(qx)\over (1-q)x},\quad q\ne 1,\;x\ne 0$$

which becomes the same as ordinary differentiation in the limit as $q\rightarrow 1.$ We shall use the *q*-analogue of the product rule

4 $$(D_qfg)(x)=f(x)(D_qg)(x)+(D_qf)(x)g(qx).$$

Exton (1977) discussed a basic analogue of the generalized Laguerre equation by means of replacing the ordinary derivatives by the *q*-operator (3) and studied some properties of certain of its solutions. Moak (1981) introduced and studied the *q*-Laguerre polynomials

5 $$L^{(\alpha )}_n(x;q)={\left(q^{\alpha +1};q\right)_n\over (q;q)_n}\sum _{k=0}^n{\left(q^{-n};q\right)_kq^{k(k-1)\over 2}\left(q^{n+\alpha +1}x\right)^k\over [k]_q!\left(q^{\alpha +1};q\right)_k},\quad \alpha >-1,n\in {\mathbb{N}}_0$$

for $0<q<1$, where $[a]_q=(1-q^a){/}(1-q)$, $(a;q)_n$ is the *q*-shifted factorial defined as

$$(a;q)_n=\prod _{k=0}^{n-1}(1-aq^k),\quad n\in {\mathbb{N}}\quad \text {with}\; (a;q)_0=1$$

and $[n]_q!$ is the *q*-factorial function defined as

$$[n]_q!=[n]_q[n-1]_q\ldots [1]_q,\quad n\in {\mathbb{N}}\quad \text {with}\; [0]_q!=1.$$

The *q*-Laguerre polynomials (5) appeared as a solution of the second order *q*-difference equation

$$xD_q^2y(x)+\left\{ [\alpha +1]_q-q^{\alpha +2}x\right\} (D_qy)(qx)+[n]_qq^{\alpha +1}y(qx)=0,\quad \alpha >-1.$$

The *q*-Laguerre polynomials has been drawn the attention of many authors who proved many properties for it. For more details see Koekoek and Swarttouw (1998), Koekoek (1992).

As a first step to extend the matrix framework of quantum calculus, the *q*-gamma and *q*-beta matrix functions have been introduced and studied in Salem (2012). Also, the basic Gauss hypergeometric matrix function has been studied in Salem (2014).

In this paper, we extend the family of *q*-Laguerre polynomials (5) of complex variables to *q*-Laguerre matrix polynomials by means of studying the solutions of the second order matrix *q*-difference equations

6 $$x(D_q^2Y)(x)+\left\{ [A+I]_q-x\lambda q^{A+2I}\right\} (D_qY)(qx)+[\alpha ]_qCY(qx)=0$$

where $\lambda ,\alpha \in {\mathbb{C}}$ and *A*, *C* and *Y*(*x*) are square matrices in ${\mathbb{C}}^{r\times r}$. The orthogonality property, explicit formula, Rodrigues-type formula, three-terms recurrence relations, generating functions and other properties will be derived.

For the sake of clarity in the presentation, we recall some properties and notations, which will be used below. Let ||*A*|| denote the norm of the matrix *A*, then the operator norm corresponding to the two-norm for vectors is

$$||A||=\max _{x\not =0}\frac{||Ax||_2}{||x||_2}=\max \left\{ \sqrt{\lambda }:\lambda \in \sigma (A^*A)\right\}$$

where $\sigma (A)$ is the spectrum of *A*: the set of all eigenvalues of *A* and $A^{*}$ denotes the transpose conjugate of *A*. If *f*(*z*) and *g*(*z*) are holomorphic functions of the complex variable *z*, which are defined in an open set $\Omega$ of the complex plane and if *A* is a matrix in ${\mathbb{C}}^{N\times N}$ such that $\sigma (A)\subset \Omega$, then from the properties of the matrix functional calculus (Dunford and Schwartz 1956), it follows that $f(A)g(A)= g(A)f(A).$

The logarithmic norm of a matrix *A* is defined as (Sastre and Defez 2006)

$$\mu (A)=\lim _{h\rightarrow 0}{\Vert I+hA\Vert -1\over h}=\max \left\{ z:z\in \sigma \left[ \left( A+A^{*}\right) /2\right] \right\} .$$

Suppose the number ${\tilde{\mu }}(A)$ such that

$$\tilde{\mu }(A)=-\mu (-A)=\min \left\{ z:z\in \sigma \left[ \left( A+A^{*}\right) /2\right] \right\} .$$

By Higueras and Garcia-Celaeta (1999), it follows that $\Vert e^{At}\Vert \le e^{t\mu (A)}$ for $t\ge 0$, we have

7 $$\begin{aligned} \Vert t^A\Vert \le \left\{ \begin{array}{ll} t^{\mu (A)},&{}\quad \text {if}\;t\ge 1,\\ t^{{\tilde{\mu }}(A)},&{}\quad \text {if}\;0\le t\le 1.\end{array} \right. \end{aligned}$$

If *A*(*k*, *n*) and *B*(*k*, *n*) are matrices on ${\mathbb{C}}^{N\times N}$ for $n,k\in {\mathbb{N}}_0$, it follows that (Defez and Jódar 1998)

8 $$\sum _{n=0}^{\infty }\sum _{k=0}^{\infty }B(k,n)=\sum _{n=0}^{\infty }\sum _{k=0}^{n}B(k,n-k)$$

and

9 $$\sum _{n=0}^{\infty }\sum _{k=0}^n B(k,n)=\sum _{n=0}^{\infty }\sum _{k=0}^\infty B(k,n+k).$$

## Matrix *q*-difference equation

The following lemmas will be used in this section.

### **Lemma 1**

*Let*$\alpha$*and*$a$*be complex number with*${\mathfrak{R}}(a)>0$*, then we have*

10 $$\lim _{x\rightarrow \infty }x^\alpha e_q(-ax)=0$$

*where*$e_q(x)$*is theq*-*analogue of the exponential function defined as*

11 $$e_q(x)=\sum _{k=0}^\infty {x^k\over [k]_q!}={1\over (x(1-q);q)_\infty },\quad |x|<(1-q)^{-1}.$$

### *Proof*

Let $f(x)=x^\alpha e_q(-ax)$. Taking the limit of logarithm of the function *f*(*x*) as $x\rightarrow \infty$ (11), gives

$$\lim _{x\rightarrow \infty }\log f(x)=\lim _{x\rightarrow \infty }\left[ \alpha \log x-\sum _{k=0}^\infty \log \left( 1+axq^k\right) \right]$$

Taking $n\in {\mathbb{N}}_0$ such that $n\ge \alpha$ yields

$$\lim _{x\rightarrow \infty }\log f(x)=\lim _{x\rightarrow \infty }\left[ \log \left( {x^\alpha \over \prod \nolimits _{k=0}^{n-1}\left( 1+axq^k\right) }\right) -\sum _{k=n}^\infty \log \left( 1+axq^k\right) \right] =-\infty$$

This means that $\lim \nolimits _{x\rightarrow \infty }f(x)=e^{-\infty }=0$ which completes the proof.

### **Lemma 2**

*Suppose that*$A\in {\mathbb{C}}^{r\times r}$*satisfying the condition*

12 $${\tilde{\mu }}(A)>-1$$

*and let*$\lambda$*be a complex number with*${\mathfrak{R}}(\lambda )>0.$*Then it follows that*

13 $$\lim _{x\rightarrow 0}\left[ x^{A+I}e_q(-qx\lambda )P_n(x)\right] =0$$

*and*

14 $$\lim _{x\rightarrow \infty }\left[ x^{A+I}e_q(-qx\lambda )P_n(x)\right] =0$$

*where*$P_n(x)$*is a matrix polynomials of degree*$n\in {\mathbb{N}}_0$.

### *Proof*

From (7), we get

$$\Vert x^{A+I}\Vert \le x^{{\tilde{\mu }}(A)+1},\quad x\rightarrow 0$$

Since $e_q(-qx\lambda )P_n(x)$ is bounded as $x\rightarrow 0$, it follows that (13) holds.

From (7), we get

$$\Vert x^{A+I}\Vert \le x^{\mu (A)+1},\quad x\rightarrow \infty$$

Let $P_n(x)=a_1x^n+a_2x^{n-1}+\cdots +a_n$ and let $0\le k\le n$, then Lemma 1 gives

$$\lim _{x\rightarrow \infty }x^{\mu (A)+k+1}e_q(-qx\lambda )=0,\quad {\mathfrak{R}}(\lambda )>0$$

it follows that

$$\lim _{x\rightarrow \infty }x^Ae_q(-qx\lambda )P_n(x)=0,\quad {\mathfrak{R}}(\lambda )>0$$

which ends the proof.

### **Theorem 3**

*Let*$m,n\in {\mathbb{N}}_0$, $A\in {\mathbb{C}}^{r\times r}$*satisfying the condition* (12) and $C\in {\mathbb{C}}^{r\times r}$*is invertible and depends on*$A$*. Let*$Y_m$*and*$Y_n$*are solutions of matrixq*-*difference equation* (6) *corresponding to*$\alpha _m$*and*$\alpha _n$*respectively, then we get*

15 $$\int _0^\infty x^Ae_q(-qx\lambda )Y_m(qx)Y_n(qx)d_qx=0,\quad m\ne n$$

*where theq-integral is the inverse ofq-derivative* (3) *defined as*

$$\int _0^\infty f(x)d_qx=(1-q)\sum _{k=-\infty }^\infty q^{k}f\left( q^k\right)$$

### *Proof*

By virtue of (4), the matrix *q*-difference equation (6) can be read as

16 $$\left( D_q\left\{ x^{A+I}e_q(-qx\lambda )(D_qY)(x)\right\} \right) (x)+[\alpha ]_qx^ACe_q(-qx\lambda )Y(qx)=0$$

Since $Y_m$ and $Y_n$ are solutions of (16) corresponding to $\alpha _m$ and $\alpha _n$ respectively, then we can easily obtain

$$\begin{aligned}&\left( [\alpha _m]_q-[\alpha _n]_q\right) x^ACe_q(-qx\lambda )Y_m(qx)Y_n(qx)\\&\quad =Y_m(qx)\left( D_q\left\{ x^{A+I}e_q(-qx\lambda )(D_qY_n)(x)\right\} \right) (x)\\&\quad \qquad -\left( D_q\left\{ x^{A+I}e_q(-qx\lambda )(D_qY_m)(x)\right\} \right) (x)Y_n(qx)\\&\quad =\left( D_q\left\{ x^{A+I}e_q(-qx\lambda )(D_qY_n)(x)Y_m(x)-x^{A+I}e_q(-qx\lambda )Y_n(x)(D_qY_m)(x)\right\} \right) (x) \end{aligned}$$

On *q*-integrating both sides from 0 to $\infty$ and by Lemma 2 and hypothesis $\alpha _m\ne \alpha _n$ yields

$$([a_m]_q-[a_n]_q)C\int _0^\infty x^Ae_q(-qx\lambda )Y_m(qx)Y_n(qx)d_qx=0,\quad m\ne n$$

which ends the proof.

Now, let us suppose that the solution of (6) has the form

17 $$Y(x)=\sum _{k=0}^\infty a_kx^k,\quad a_k\in {\mathbb{C}}^{r\times r},\quad a_0\ne 0_{r\times r}$$

where $0_{r\times r}$ is the null matrix in ${\mathbb{C}}^{r\times r}$.

To determine the matrices $a_k$. Taking formal *q*-derivatives (3) of $Y(x)$, it follows that

$$(D_qY)(x)=\sum _{k=0}^\infty [k+1]_qa_{k+1}x^k,\quad \text {and}\quad \left( D^2_qY\right) (x)=\sum _{k=0}^\infty [k+1]_q[k+2]_qa_{k+2}x^k$$

Substituting into (6) would yield

$$\begin{aligned}&\sum _{k=0}^\infty [k+1]_q[k+2]_qa_{k+2}x^{k+1}+\left( [A+I]_q-x\lambda q^{A+2I}\right) \sum _{k=0}^\infty [k+1]_qa_kq^kx^{k}\\&\quad +[\alpha ]_qC\sum _{k=0}^\infty a_{k}q^kx^k=0 \end{aligned}$$

Equating the coefficients of $x^k$, $k\in {\mathbb{N}}_0$ would yield

$$[A+I]_qa_1+[\alpha ]_qCa_0=0$$

and

$$\left( [k]_q+[A+I]_qq^k\right) [k+1]_qa_{k+1}-q^k\left( \lambda [k]_qq^{A+I}-[\alpha ]_qC\right) a_k=0,\quad k\in {\mathbb{N}}$$

which can be read as

18 $$[A+kI]_q[k]_qa_{k}-q^{k-1}\left( \lambda [k-1]_qq^{A+I}-[\alpha ]_qC\right) a_{k-1}=0,\quad k\in {\mathbb{N}}$$

For existence of the second order *q*-difference equation, we seek the sufficient condition

$$C=\lambda q^{A+I},\quad \text {and}\quad \alpha =n\qquad \hbox {for some non-negative integer } n$$

We have to suppose that $q^{-k}\not \in \sigma (q^A)$, $k\in {\mathbb{N}}_0$ to ensure that the relevant $(I-q^{A+kI})$ exists. Therefore, (18) gives

$$a_k={\lambda [k-n-1]_q\over [k]_q}[A+kI]_q^{-1}q^{A+(n+1)I}a_{k-1},\quad k\in {\mathbb{N}}$$

which leads to

$$a_k={\left( q^{-n};q\right) _kq^{\left( \begin{array}{c}k\\ 2\end{array}\right) }\lambda ^k \over [k]_q!}\left( q^{A+I};q\right) _k^{-1}q^{(A+(n+1)I)k}a_0$$

Letting the boundary condition $Y(0)={(q^{A+I};q)_n\over (q;q)_n}$ which reveals that $a_0={(q^{A+I};q)_n\over (q;q)_n}$ and so we can seek the following definition for the *q*-Laguerre matrix function which verified the Eq. (6)

### **Definition 4**

Let $n\in {\mathbb{N}}_0$, $\lambda$ be a complex number with ${\mathfrak{R}}(\lambda )>0$ and $A\in {\mathbb{C}}^{r\times r}$ satisfying the conditions (12) and $q^{-k}\not \in \sigma (q^A)$ for all $0\le k\le n$. The *q*-Laguerre matrix polynomials can be defined as

19 $$\begin{aligned} L_n^{(A,\lambda )}(x;q)=\sum _{k=0}^n{\left( q^{-n};q\right) _kq^{\left( \begin{array}{c}k\\ 2\end{array}\right) }\lambda ^kx^k\over [k]_q!(q;q)_n}\left( q^{A+I};q\right) _k^{-1}\left( q^{A+I};q\right) _nq^{(A+(n+1)I)k}. \end{aligned}$$

### *Remark 5*

When letting $q\rightarrow 1$, the matrix *q*-difference equation (6) tends to the matrix differential equation (1) and also the *q*-Laguerre matrix polynomials (19) approache to the Laguerre matrix polynomials (2). We proved that the *q*-Laguerre matrix polynomials (19) hold for ${\tilde{\mu }}(A)>-1$ but Jódar et al. (1994) proved that the Laguerre matrix polynomials (2) hold for ${\mathfrak{R}}(z)>-1$ for all $z\in \sigma (A)$ which equivalently $\beta (A)>-1$ where $\beta =\min \{{\mathfrak{R}}(z):z\in \sigma (A)\}$. It is worth noting that the important relation between $\beta (A)$ and ${\tilde{\mu }}(A)$, which states $\beta (A)>{\tilde{\mu }}(A)$ (Salem 2012). Therefore, the definition of the Laguerre matrix polynomials (2) can be extended for ${\tilde{\mu }}(A)>-1$.

## Generating functions

The basic hypergeometric series is defined as Gasper and Rahman (2004)

20 $$\begin{aligned} {}_r\phi _s\left[ ^{a_1,a_2,\ldots ,a_r}_{b_1,b_2,\ldots ,b_s};q,z\right]&={}_r\phi _s\left( a_1,a_2,\ldots ,a_r;b_1,b_2,\ldots ,b_s;q,z\right) \nonumber \\&=\sum _{n=0}^\infty \frac{\left( a_1,a_2,\ldots ,a_r;q\right) _n}{\left( q,b_1,b_2,\ldots ,b_s;q\right) _n}\left( (-1)^nq^{\left( \begin{array}{c}n\\ 2\end{array}\right) }\right) ^{s-r+1}z^n, \end{aligned}$$

for all complex variable *z* if $r\le s,0<|q|<1$ and for $|z|<1$ if $r=s+1$, where

$$\left( a_1,a_2,\ldots ,a_r;q\right) _n=(a_1;q)_n(a_2;q)_n\ldots (a_r;q)_n$$

Notice that the *q*-shifted function $(a;q)_n$ has the summation Koekoek and Swarttouw (1998)

21 $$(a;q)_n=\sum _{k=0}^n{n\brack k}_qq^{\left( \begin{array}{c}k\\ 2\end{array}\right) }(-a)^k$$

where ${n\brack k}_q$ is the *q*-binomial coefficients defined as

22 $$\begin{aligned} {n\brack k}_q={(q;q)_n\over (q;q)_k(q;q)_{n-k}}={\left( q^{-n};q\right) _k\over (q;q)_k}(-1)^kq^{kn-{\left( \begin{array}{c}n\\ 2\end{array}\right) }}. \end{aligned}$$

Also it has the well-known identities

23 $$(a;q)_{n-k}={(a;q)_n\over (q^{1-n}a^{-1};q)_k}\left( -{q\over a}\right) ^kq^{{\left( \begin{array}{c}k\\ 2\end{array}\right) }-nk},\quad a\ne 0,\quad k=0,1,2,\ldots n$$

and

24 $$(a;q)_n={(a;q)_\infty \over \left( aq^n;q\right) _\infty },\quad n\in {\mathbb{N}}_0.$$

The *q*-shifted factorial matrix function was defined in Salem (2012) as

25 $$(A;q)_0=I,\quad (A;q)_n=\prod _{k=0}^{n-1}\left( I-Aq^k\right) ,\quad n\in {\mathbb{N}}$$

and satisfies

26 $$\begin{aligned} (A;q)_\infty =\lim _{n\rightarrow \infty }\prod _{k=0}^{n-1}\left( I-Aq^k\right) =\prod _{k=0}^\infty \left( I-Aq^k\right) =\sum _{k=0}^\infty {(-1)^kq^{\left( \begin{array}{c}k\\ 2\end{array}\right) }\over (q;q)_k}A^k. \end{aligned}$$

Furthermore, if $\Vert A\Vert <1$ and $q^{-k}\not \in \sigma (A)$, $k\in {\mathbb{N}}_0$, the infinite product (26) converges invertibly and

27 $$(A;q)_\infty ^{-1}=\sum _{k=0}^\infty {A^k\over (q;q)_k}.$$

In Salem (2012) a proof of the matrix *q*-binomial theorem can be found

28 $$\sum _{k=0}^\infty {(B;q)_k\over (q;q)_k}A^k=(A;q)_\infty ^{-1}(AB;q)_\infty ,$$

for all commutative matrices $A,B\in {\mathbb{C}}^{r\times r}$ and $q^{-k}\not \in \sigma (A)$, $k\in {\mathbb{N}}_0$.

For complete this section, we need the following:

### **Lemma 6**

*Let*$A$* be a square matrix in*${\mathbb{C}}^{r\times r}$*and*$n\in {\mathbb{N}}_0$*. Then, we have the following three identities*

- 29 $$\begin{aligned} (A;q)_n=(A;q)_\infty \left( Aq^n;q\right) _\infty ^{-1}=\sum _{k=0}^n{n\brack k}_qq^{\left( \begin{array}{c}k\\ 2\end{array}\right) }(-A)^k,\quad \Vert A\Vert <1 \end{aligned}$$
  *where*$q^{-m}\not \in \sigma (A),m=n,n+1,\ldots,$*and its reciprocal*
- 30 $$(A;q)_n^{-1}=(A;q)_\infty ^{-1}\left( Aq^n;q\right) _\infty =\sum _{k=0}^\infty {(q^n;q)_k\over (q;q)_k}A^k,\quad \Vert A\Vert <1$$
  *where*$q^{-k}\not \in \sigma (A),k\in {\mathbb{N}}_0$*. Also the identity*
- 31 $$\left( Aq^k;q\right) _{n-k}=(A;q)_k^{-1}(A;q)_n,\quad k=0,1,\ldots ,n$$
  *holds for*$$q^{-r}\not \in \sigma (A),r=0,1,\ldots ,k.$$

### *Proof*

Let $\Vert A\Vert <1$ and $q^{-m}\not \in \sigma (A),m=n,n+1,\ldots$, then we have

$$\begin{aligned} (A;q)_n&= {} \prod _{k=0}^{n-1}\left( 1-Aq^k\right) \prod _{k=n}^\infty \left( 1-Aq^k\right) \left( 1-Aq^k\right) ^{-1}\\&= {} \prod _{k=0}^\infty \left( 1-Aq^k\right) \prod _{k=n}^\infty \left( 1-Aq^k\right) ^{-1}\\&= {} (A;q)_\infty \prod _{k=0}^\infty \left( 1-Aq^{k+n}\right) ^{-1}=(A;q)_\infty \left( Aq^n;q\right) _\infty ^{-1} \end{aligned}$$

Using (28) and (23) yields (29). The relation (30) comes immediately from (29) and (28). Also (31) can be easily obtained.

### **Lemma 7**

*Let*$A$*and*$C$*are matrices in*${\mathbb{C}}^{r\times r}$*such that*$q^{-n}\not \in \sigma (C)$*for all*$n\in {\mathbb{N}}_0$*and*$a\in {\mathbb{C}},$*the matrix functions*

32 $${}_0\phi _1(-;C;q,A)=\sum _{n=0}^\infty {q^{n(n-1)}\over (q;q)_n}(C;q)_n^{-1}A^n$$

*and*

33 $${}_0\phi _2(-;C,a;q,A)=\sum _{n=0}^\infty {(-1)^nq^{3n(n-1)\over 2}\over (q;q)_n(a;q)_n}(C;q)_n^{-1}A^n$$

*converge absolutely*.

### *Proof*

The condition $q^{-n}\not \in \sigma (C)$ guarantees that $I-Cq^n$ is invertible for all integer $n\ge 0$. Now take *n* large enough so that $\Vert C\Vert <|q|^{-n}$, by the perturbation lemma (Constantine and Muirhead 1972)

34 $$\Vert A^{-1}-B^{-1}\Vert \le {\Vert B^{-1}\Vert ^2\Vert A-B\Vert \over 1-\Vert B^{-1}\Vert \Vert A-B\Vert },$$

one gets

35 $$\begin{aligned} \Vert \left( I-q^nC\right) ^{-1}\Vert&=\Vert \left( I-q^nC\right) ^{-1}-I+I\Vert \nonumber \\&\le \Vert \left( I-q^nC\right) ^{-1}-I\Vert +1\nonumber \\&\le {q^n\Vert C\Vert \over 1-q^n\Vert C\Vert }+1\nonumber \\&={1\over 1-q^n\Vert C\Vert },\quad n>-{\ln \Vert C\Vert \over \ln |q|}. \end{aligned}$$

If we take

$$\begin{aligned} F(n)=\left\{ \begin{array}{ll} 1,&{}\quad n=0,\\ \prod \nolimits _{k=0}^{n-1}\Vert \left( I-q^{k}C\right) ^{-1}\Vert ,&{}\quad n\ge 1,\end{array}\right. \end{aligned}$$

and by the relation (35), we get

$$\Vert {}_0\phi _1(-;C;q,A)\Vert \le \sum _{n=0}^\infty \left\| {q^{n(n-1)}(C;q)_n^{-1}\over (q;q)_n}A^n\right\| \le \sum _{n=0}^\infty {q^{n(n-1)}F(n)\over (q;q)_n}\Vert A\Vert ^n.$$

Using the ratio test and the perturbation lemma (34), one finds

$$\begin{aligned}&\lim _{n\rightarrow \infty }\left| {q^{n(n+1)}F(n+1)(q;q)_n\Vert A\Vert ^{n+1}\over q^{n(n-1)}F(n)(q;q)_{n+1}\Vert A\Vert ^n}\right| \\&\quad =\lim _{n\rightarrow \infty }{\Vert \left( I-q^nC\right) ^{-1}\Vert \over 1-q^{n+1}}q^{2n}\Vert A\Vert \\&\quad \le \lim _{n\rightarrow \infty }{q^{2n}\over \left( 1-q^{n+1}\right) \left( 1-q^n\Vert C\Vert \right) }\Vert A\Vert =0. \end{aligned}$$

Thus, the matrix power series (32) is absolutely convergent. Similarly, (33) can be proved. This ends the proof.

Since the function ${}_1\phi _1(a;0;q,z)$ is analytic for all complex numbers *a* and *z*, the matrix functional calculus tells that the matrix function ${}_1\phi _1(a;0;q,A)$ is also convergent for all complex number *a* and for all matrices $A\in {\mathbb{C}}^{r\times r}$.

### **Lemma 8**

*Let*$A$*be matrix in*${\mathbb{C}}^{r\times r}$*such that*$q^{-n}\not \in \sigma (C)$*for all*$n\in {\mathbb{N}}_0$*and a be a complex number. We have the transformation*

36 $${}_1\phi _1(a;0;q,A)=(A;q)_\infty {}_0\phi _1(-;A;q,aA)$$

### *Proof*

The relation (21) can be exploited to prove the transformation

$$\begin{aligned} {}_1\phi _1(a;0;q,A)&=\sum _{n=0}^\infty {(-1)^nq^{\left( \begin{array}{c}n\\ 2\end{array}\right) }(a;q)_n\over (q;q)_n}A^n&=\sum _{n=0}^\infty {(-1)^nq^{\left( \begin{array}{c}n\\ 2\end{array}\right) }\over (q;q)_n}A^n\sum _{k=0}^n{n\brack k}_qq^{\left( \begin{array}{c}k\\ 2\end{array}\right) }(-a)^k \end{aligned}$$

Using the relations (9) and (26) lead to

$$\begin{aligned} {}_1\phi _1(a;0;q,A)&=\sum _{n=0}^\infty \sum _{k=0}^\infty {n+k\brack k}_q{(-1)^{n+k}q^{{\left( \begin{array}{c}k\\ 2\end{array}\right) }+{\left( \begin{array}{c}n+k\\ 2\end{array}\right) }}(-a)^k\over (q;q)_{n+k}}A^{n+k}\\&=\sum _{k=0}^\infty {q^{k(k-1)}a^k\over (q;q)_k}A^k\sum _{n=0}^\infty {(-1)^nq^{{\left( \begin{array}{c}n\\ 2\end{array}\right) }+nk}\over (q;q)_n}A^n\\&=\sum _{k=0}^\infty {q^{k(k-1)}a^k\over (q;q)_k}A^k(Aq^k;q)_\infty \end{aligned}$$

Inserting the relation (30) into the above relation gives

$${}_1\phi _1(a;0;q,A)=(A;q)_\infty \sum _{k=0}^\infty {q^{k(k-1)}a^k\over (q;q)_k}A^k(A;q)_k^{-1}=(A;q)_\infty {}_0\phi _1(-;A;q,aA)$$

This ends the proof.

### **Theorem 9**

*Let*$$n\in {\mathbb{N}}_0,$$$$\lambda$$*be a complex number with*$${\mathfrak{R}}(\lambda )>0$$*and*$$A\in {\mathbb{C}}^{r\times r}$$*satisfying the conditions* (12) *and*$$q^{-k}\not \in \sigma (q^A)$$*for all*$$k\in {\mathbb{N}}_0$$.* Theq-Laguerre matrix polynomials have the generating functions*

37 $${1\over (t;q)_\infty }{}_1\phi _1\left( -\lambda x(1-q);0;q,q^{A+I}t\right) =\sum _{n=0}^\infty L_n^{(A,\lambda )}(x;q)t^n,$$

38 $$(t;q)_\infty \left( tq^{-A-I};q\right) _\infty ^{-1}{}_0\phi _1\left( -;t;q,-\lambda tx(1-q)\right) =\sum _{n=0}^\infty q^{-n(A+I)}L_n^{(A,\lambda )}(x)t^n$$

39 $${1\over (t;q)_\infty }{}_0\phi _1\left( -;q^{A+I};q,-q^{A+I}(1-q)\lambda xt\right) =\sum _{n=0}^\infty \left( q^{A+I};q\right) _n^{-1}L_n^{(A,\lambda )}(x;q)t^n$$

*for all*$|t|<1$*and*

40 $$(t;q)_\infty {}_0\phi _2\left( -;q^{A+I},t;q,-\lambda tx(1-q)q^{A+I}\right) =\sum _{n=0}^\infty (-1)^nq^{\left( \begin{array}{c}n\\ 2\end{array}\right) }\left( q^{A+I};q\right) _n^{-1}L_n^{(A,\lambda )}(x)t^n$$

*for all*$$t\in {\mathbb{C}}$$.

### *Proof*

The left hand side of (37) can be rewritten by means of using the transformation (36) as follows

$$\begin{aligned} LHS&={1\over (t;q)_\infty }{}_1\phi _1\left( -\lambda x(1-q);0;q,q^{A+I}t\right) \\&={1\over (t;q)_\infty }\left( tq^{A+I};q\right) _\infty {}_0\phi _1\left( -;tq^{A+I};q,-\lambda xt(1-q)q^{A+I}\right) \\&={1\over (t;q)_\infty }\left( tq^{A+I};q\right) _\infty \sum _{n=0}^\infty {(-1)^nq^{n(n-)}\lambda ^nx^nt^n\over [n]_q!}\left( tq^{A+I};q\right) _n^{-1}q^{n(A+I)},\quad |t|<1 \end{aligned}$$

Using (30) followed by (28) give

$$\begin{aligned} LHS&=\sum _{n=0}^\infty {(-1)^nq^{n(n-1)}\lambda ^nx^nt^n\over [n]_q!}{\left( tq^{A+(n+1)I};q\right) _\infty \over (t;q)_\infty }q^{n(A+I)}\\&=\sum _{n=0}^\infty {(-1)^nq^{n(n-1)}\lambda ^nx^nt^n\over [n]_q!}\sum _{k=0}^\infty {\left( q^{A+(n+1)I};q\right) _k\over (q;q)_k}t^kq^{n(A+I)} \end{aligned}$$

In view of (8), we get

$$LHS=\sum _{n=0}^\infty t^n\sum _{k=0}^n{(-1)^kq^{k(k-1)}\lambda ^kx^k\over [k]_q!(q;q)_{n-k}}\left( q^{A+(k+1)I};q\right) _{n-k}q^{k(A+I)}$$

Inserting (23) and (31) with taking into account the definition of *q*-Laguerre matrix polynomials (19) to obtain the right hand side of (37). Similarly, we can find (38) with noting that the convergence of $(tq^{-A-I};q)_\infty ^{-1}$ needs $\Vert tq^{-A-I}\Vert <1$ which is equivalent to $|t|<\Vert q^{A+I}\Vert \le q^{{\tilde{\mu }}(A)+1}<1$. In order to prove (39), we have

$$\begin{aligned}&{1\over (t;q)_\infty }{}_0\phi _1\left( -;q^{A+I};q,-q^{A+I}(1-q)\lambda xt\right) \\&\quad =\sum _{n=0}^\infty {t^n\over (q;q)_n}\sum _{n=0}^\infty {(-1)^nq^{n(n-1)}\lambda ^nx^nt^n\over [n]_q!}\left( q^{A+I};q\right) _n^{-1}q^{(A+I)n}\\&\quad =\sum _{n=0}^\infty t^n\sum _{k=0}^n{(-1)^kq^{k(k-1)}\lambda ^kx^k\over [k]_q!(q;q)_{n-k}}\left( q^{A+I};q\right) _k^{-1}q^{(A+I)k} \end{aligned}$$

Using the well-known identity (23) to obtain (39). (40) is similar to (37) and (38).

### **Corollary 10**

*Let*$$n\in {\mathbb{N}}_0,$$$$\lambda$$*be a complex number with*$${\mathfrak{R}}(\lambda )>0$$*and*$$A\in {\mathbb{C}}^{r\times r}$$*satisfying the conditions* (12) *and*$$q^{-k}\not \in \sigma (q^A)$$*for all*$$0\le k\le n.$$*Theq-Laguerre matrix polynomials can be defined as*

41 $$L_n^{(A,\lambda )}(x;q)=\sum _{k=0}^n{(q^{-n};q)_k(-\lambda x(1-q);q)_k\over (q;q)_k}q^{(A+(n+1)I)k}.$$

### *Proof*

The generating function (37) can be expanded as

$$\begin{aligned}&{1\over (t;q)_\infty }{}_1\phi _1\left( -\lambda x(1-q);0;q,q^{A+I}t\right) \\&\quad =\sum _{n=0}^\infty {t^n\over (q;q)_n}\sum _{n=0}^\infty {(-1)^nq^{\left( \begin{array}{c}n\\ 2\end{array}\right) }(-\lambda x(1-q);q)_n\over (q;q)_n}q^{(A+I)n}t^n\\&\quad =\sum _{n=0}^\infty t^n\sum _{n=0}^n{(-1)^kq^{\left( \begin{array}{c}k\\ 2\end{array}\right) }(-\lambda x(1-q);q)_k\over (q;q)_k(q;q)_{n-k}}q^{(A+I)k}\\&\quad =\sum _{n=0}^\infty t^n\sum _{n=0}^n{(q^{-n};q)_k(-\lambda x(1-q);q)_k\over (q;q)_k(q;q)_{n}}q^{(A+(n+1)I)k}\\&\quad =\sum _{n=0}^\infty L_n^{(A,\lambda )}(x;q)t^n. \end{aligned}$$

This ends the proof.

### *Remark 11*

In view of the explicit expressions of the *q*-Laguerre matrix polynomials (19) and (41), with replacing $q^{A+I}$ and $-\lambda x(1-q)$ by *A* and *x*, respectively, we can derive the matrix transformation

42 $${}_2\phi _1\left( q^{-n},x;0;q,Aq^n\right) =(A;q)_n{}_1\phi _1\left( q^{-n};A;q,xAq^n\right)$$

which tends to the transformation (36) as $n\rightarrow \infty$.

## Recurrence relations and Rodrigues-type formula

This section is devoted to introduce some recurrence relations and Rodrigues type formula for the *q*-Laguerre matrix polynomials.

### **Theorem 12**

*Let*$\lambda$*be a complex number with*${\mathfrak{R}}(\lambda )>0$*and*$A\in {\mathbb{C}}^{r\times r}$*satisfying the conditions* (12) *and*$q^{-k}\not \in \sigma (q^A)$*for all*$0\le k\le n.$*Then, the q-Laguerre matrix polynomials satisfy the three-term matrix recurrence relation*

43 $$\begin{aligned}&[n+1]_qL_{n+1}^{(A,\lambda )}(x;q)+\left( \lambda xq^{A+(2n+1)I}-q[n]_q-[A+(n+1)I]_q\right) L_n^{(A,\lambda )}(x;q)\nonumber \\&\quad \qquad +[A+nI]_qL_{n-1}^{(A,\lambda )}(x;q)=0,\quad n\in {\mathbb{N}}. \end{aligned}$$

### *Proof*

Let the matrix-valued function

44 $$F(x,t,A)={1\over (t;q)_\infty }{}_1\phi _1\left( -\lambda x(1-q);0;q,q^{A+I}t\right)$$

Using Jackson *q*-derivatives operator (3) and the *q*-analogue of the product rule (4) give

$$\begin{aligned} (D_qF)(t)&={1\over (1-q)(t;q)_\infty }{}_1\phi _1\left( -\lambda x(1-q);0;q,q^{A+I}t\right) \\&\quad -{q^{A+I}\over (1-q)(qt;q)_\infty }\left\{ {}_1\phi _1\left(-\lambda x(1-q);0;q,q^{A+I}qt\right)\right.\\ &\left.\quad +\lambda x(1-q){}_1\phi _1\left( -\lambda x(1-q);0;q,q^{A+I}q^2t\right) \right\} \end{aligned}$$

Inserting the above relation into the generating function (37) with taking the *q*-derivative of the right hand side yields

$$\begin{aligned}&\sum _{n=0}^\infty \left( 1-q^{A+(n+1)I}\right) L_n^{(A,\lambda )}(x;q)t^n-q\sum _{n=0}^\infty \left( 1-q^{A+(n+1)I}\right) L_n^{(A,\lambda )}(x;q)t^{n+1}\\&\qquad -\lambda x(1-q)q^{A+I}\sum _{n=0}^\infty L_n^{(A,\lambda )}(x;q)q^{2n}t^n\\&\quad =\sum _{n=0}^\infty (1-q^{n+1})L_{n+1}^{(A,\lambda )}(x;q)t^n-q\sum _{n=0}^\infty (1-q^{n+1})L_{n+1}^{(A,\lambda )}(x;q)t^{n+1} \end{aligned}$$

Equating to the zero matrix the coefficient of each power $t^n$ it follows that

$$L_1^{(A,\lambda )}(x;q)=\left( [A+I]_q-\lambda xq^{A+I}\right) L_0^{(A,\lambda )}(x;q)$$

and

$$\begin{aligned}&\left( 1-q^{A+(n+1)I}\right) L_n^{(A,\lambda)}(x;q)-\left( 1-q^{A+nI}\right) qL_{n-1}^{(A,\lambda)}(x;q)\\ &\quad\qquad-\lambda x(1-q)q^{A+(2n+1)I}L_n^{(A,\lambda )}(x;q)\\&\quad \quad\qquad =\left( 1-q^{n+1}\right) L_{n+1}^{(A,\lambda )}(x;q)-\left( 1-q^{n}\right) qL_{n}^{(A,\lambda )}(x;q). \end{aligned}$$

Therefore the *q*-Laguerre matrix polynomials satisfy the three-term matrix recurrence relation (43).

### **Theorem 13**

*Let*$\lambda$*be a complex number with*${\mathfrak{R}}(\lambda )>0$*and*$A\in {\mathbb{C}}^{r\times r}$*satisfying the conditions* (12) *and*$q^{-k}\not \in \sigma (q^A)$*for all*$0\le k\le n.$*Then, the q-Laguerre matrix polynomials satisfy the matrix relation*

45 $$\sum _{i=0}^nq^iL_{i}^{(A,\lambda )}(x;q)=L_{n}^{(A+I,\lambda )}(x;q),\quad n\in {\mathbb{N}}_0.$$

### *Proof*

It is not difficult, by using (19) to see that the *q*-Laguerre matrix polynomials satisfy the forward shift operator

$$L_{n}^{(A,\lambda )}(x;q)-L_{n}^{(A,\lambda )}(qx;q)=-\lambda x(1-q)q^{A+I}L_{n-1}^{(A+I,\lambda )}(qx;q)$$

which is equivalent to

$$D_qL_{n}^{(A,\lambda )}(x;q)=-\lambda q^{A+I}L_{n-1}^{(A+I,\lambda )}(qx;q).$$

By iteration this process *k*-times, we can get the relation

$$D_q^kL_{n}^{(A,\lambda )}(x;q)=(-1)^k\lambda ^kq^{k(A+kI)}L_{n-k}^{(A+kI,\lambda )}(xq^k;q),k=0,1,\ldots ,n;\quad n\in {\mathbb{N}}_0.$$

When $k=n$, we obtain

$$D_q^nL_{n}^{(A,\lambda )}(x;q)=(-1)^n\lambda ^nq^{n(A+nI)},\quad n\in {\mathbb{N}}_0$$

which can be also obtained from *n*th term of (19) with fact that $D_q^nx^n=[n]_q!$. It is easy to show that

$$(1-t)F(x,t,A+I)=F(x,qt,A)$$

From (37), we can deduce that

$$\sum _{n=0}^\infty L_{n}^{(A+I,\lambda )}(x;q)t^n-\sum _{n=0}^\infty L_{n}^{(A+I,\lambda )}(x;q)t^{n+1}=\sum _{n=0}^\infty q^nL_{n}^{(A,\lambda )}(x;q)t^n$$

which gives

$$q^nL_{n}^{(A,\lambda )}(x;q)=L_{n}^{(A+I,\lambda )}(x;q)-L_{n-1}^{(A+I,\lambda )}(x;q).$$

In view of iteration the above formula, we get the desired result.

In order to obtain the Rodrigues-type formula for the *q*-Laguerre matrix polynomials, we derive the following theorem.

### **Theorem 14**

(Rodrigues-type formula) *Let*$\lambda$*be a complex number with*${\mathfrak{R}}(\lambda )>0$*and*$A\in {\mathbb{C}}^{r\times r}$*satisfying the conditions* (12) *and*$q^{-k}\not \in \sigma (q^A)$*for all*$0\le k\le n.$*Then, the Rodrigues-type formula for the q-Laguerre matrix polynomials can be provided as*

46 $$[n]_q!x^Ae_q(-\lambda x)L_n^{(A,\lambda )}(x;q)=D_q^n\left\{ x^{A+nI}e_q(-\lambda x)\right\} ,\quad n\in {\mathbb{N}}_0.$$

### *Proof*

Using (3) yields

$$D_q^ke_q(-x\lambda )=(-1)^k\lambda ^ke_q(-\lambda x)$$

and

$$\begin{aligned} D_q^{n-k}x^{A+nI}&=[A+nI]_q[A+(n-1)I]_q\ldots [A+(k+1)I]_qx^{A+kI}\\&={\left( q^{A+(k+1)I};q\right) _{n-k}\over (1-q)^{n-k}}x^{A+kI} \end{aligned}$$

which can be rewrite by using (31) as

$$D_q^{n-k}x^{A+nI}={\left( q^{A+I};q\right) _{k}^{-1}\left( q^{A+I};q\right) _{n}\over (1-q)^{n-k}}x^{A+kI}.$$

From the Leibniz’s rule for the *n*th *q*-derivative of a product rule Koekoek and Swarttouw (1998)

$$D_q^n\{f(x)g(x)\}=\sum _{k=0}^n{n\brack k}_q\left( D_q^{n-k}f\right) \left( xq^k\right) \left( D_q^kg\right) (x),\quad n\in {\mathbb{N}}_0$$

and the properties of the matrix functional calculus, it follows that

$$D_q^n\left\{ x^{A+nI}e_q(-\lambda x)\right\} =x^Ae_q(-\lambda x)\sum _{k=0}^n(-1)^k{n\brack k}_q{\left( q^{A+I};q\right) _{k}^{-1}\left( q^{A+I};q\right) _{n}\over (1-q)^{n-k}}q^{kA+k^2I}\lambda ^kx^k.$$

Inserting the last side of relation (22) to obtain the Rodrigues-type formula for the *q*-Laguerre matrix polynomials.

## Orthogonality property

Suppose that the inner product $\langle f,g\rangle$ for a suitable two matrix-valued functions *f* and *g* is defined as

47 $$\langle f,g\rangle =\int _0^\infty x^Ae_q(-\lambda x)f(x)g(x)d_qx$$

Let $P_n(x)$ be a matrix polynomials for $n\ge 0$. We say that the sequence $\{P_n(x)\}_{n\ge 0}$ is an orthogonal matrix polynomials sequence with respect to the inner product $\langle ,\rangle$ provided for all nonnegative integers $n$ and $m$

1. $P_n(x)$ is a matrix polynomial of degree *n* with non-singular leader coefficient.
2. $$\langle P_n(x),P_m(x)\rangle =0$$ for all $$n\ne m$$.
3. $\langle P_n(x),P_n(x)\rangle$ is invertible for $n\ge 0$.

Let us assume that $n\in {\mathbb{N}}_0$, $\lambda$ be a complex number with ${\mathfrak{R}}(\lambda )>0$ and $A\in {\mathbb{C}}^{r\times r}$ satisfying the conditions (12) and $q^{-k}\not \in \sigma (q^A)$ for all $0\le k\le n$, then the first and second conditions for the orthogonality of the *q*-Laguerre matrix polynomials come from Definition 4 and Theorem 3. The second condition, upon the inner product, states

48 $$\left\langle L_n^{(A,\lambda )}(x;q),L_m^{(A,\lambda )}(x;q)\right\rangle =0, \quad n\ne m$$

### **Lemma 15**

*Let*$$n\in {\mathbb{N}}_0,$$$$\lambda$$*be a complex number with*$${\mathfrak{R}}(\lambda )>0$$*and*$$A\in {\mathbb{C}}^{r\times r}$$*satisfying the conditions* (12) *and*$$q^{-k}\not \in \sigma (q^A)$$*for all*$$k\in {\mathbb{N}}_0.$$*Then, we get*

49 $$\begin{aligned} \left\langle L_n^{(A,\lambda )}(x;q),L_n^{(A,\lambda )}(x;q)\right\rangle ={q^{\left( \begin{array}{c}n\\ 2\end{array}\right) }\lambda ^n\over [n]_q!}q^{nA}I_0(A+nI) \end{aligned}$$

*where*

50 $$I_0(A+nI)=\int _0^\infty x^{A+nI}e_q(-\lambda x)d_qx$$

### *Proof*

Since the *q*-Laguerre matrix polynomials $L_n^{(A,\lambda )}(x;q)$ are polynomials of degree *n* with a non-singular leader coefficient, then there exist constants matrices $C_k, k=0,1,2,\ldots , n$ such that

51 $$\sum _{k=0}^nC_kL_k^{(A,\lambda )}(x;q)=x^nI$$

where *I* is the identity matrix in ${\mathbb{C}}^{r\times r}$. In view of (48) and (51), we can deduce that

$$\left\langle x^k,L_n^{(A,\lambda )}(x;q)\right\rangle =0, \quad k=0,1,2,\ldots ,n-1.$$

Thus from (19), we obtain

52 $$\left\langle L_n^{(A,\lambda )}(x;q),L_n^{(A,\lambda )}(x;q)\right\rangle ={(-1)^n\lambda ^nq^{n(A+nI)}\over [n]_q!}\left\langle x^n,L_n^{(A,\lambda )}(x;q)\right\rangle$$

To evaluate the inner product $\langle x^n,L_n^{(A,\lambda )}(x;q)\rangle$, let us suppose that

$$I_n(A)=\int _0^\infty x^{A+nI}e_q(-\lambda x)L_n^{(A,\lambda )}(x;q)d_qx,\quad n\in {\mathbb{N}}_0.$$

Inserting the Rodrigues-type formula for the *q*-Laguerre matrix polynomials (46) yields

$$[n]_q!I_n(A)=\int _0^\infty x^{n}D_q^n\left\{ x^{A+nI}e_q(-\lambda x)\right\} d_qx.$$

On *q*-integrating by parts, which states

$$\int _0^\infty f(x)(D_qg)(x)d_qx=\{f(x)g(x)\}_0^\infty -\int _0^\infty g(qx)D_qf(x)d_qx,$$

we find

$$\begin{aligned}{}[n]_q!I_n(A)&=\left\{ x^nD_q^{n-1}\left\{ x^{A+nI}e_q(-\lambda x)\right\} \right\} _0^\infty \\&\quad -[n]_q\int _0^\infty x^{n-1}\left( D_q^{n-1}\left\{ x^{A+nI}e_q(-\lambda x)\right\} \right) (qx)d_qx \end{aligned}$$

which can be rewritten by Rodrigues-type formula (46) as

$$\begin{aligned}{}[n]_q!I_n(A)&=[n-1]_q!\left\{ x^{A+(n+1)I}e_q(-\lambda x)L_{n-1}^{(A+I,\lambda )}(x;q)\right\} _0^\infty \\&\quad -[n]_q!q^{A+I}\int _0^\infty x^{A+nI}e_q(-\lambda qx)L_{n-1}^{(A+I,\lambda )}(qx;q)d_qx. \end{aligned}$$

By using Lemma 2, we obtain

$$I_n(A)=-q^{A+I}\int _0^\infty x^{A+nI}e_q(-\lambda qx)L_{n-1}^{(A+I,\lambda )}(qx;q)d_qx.$$

Using the *q*-analogue of the integration theorem by change of variable from *qx* to *x* yields

$$I_n(A)=-q^{-n}\int _0^\infty x^{A+nI}e_q(-\lambda x)L_{n-1}^{(A+I,\lambda )}(x;q)d_qx=-q^{-n}I_{n-1}(A+I)$$

which leads to

$$I_n(A)=(-1)^nq^{-{n(n+1)\over 2}}I_0(A+nI).$$

Hence, from (52), we have the desired results.

### **Theorem 16**

*Let us assume that*$A\in {\mathbb{C}}^{r\times r}$*satisfying the condition*${\tilde{\mu }}(A)>0,$*then we have*

53 $$\Gamma _q(A)=K_q(A,\lambda )\int _0^\infty x^{A-I}e_q(-\lambda x)d_qx$$

*where*$\Gamma _q(A)$*is the q-gamma matrix function defined by* Salem (2012) *as*

54 $$\Gamma _q(A)=\int _0^{1\over 1-q}x^{A-I}E_q(-xq)d_qx$$

*and*

55 $$K_q(A,\lambda )=(1-q)^{I-A}(-\lambda (1-q);q)_\infty \left( {-q\over \lambda (1-q)};q\right) _\infty e_q(-\lambda q^A)\left( {-q^{I-A}\over \lambda (1-q)};q\right) _\infty$$

### *Proof*

Let the function

$${\tilde{\Gamma }}_q(A)=\int _0^\infty x^{A-I}e_q(-\lambda x)d_qx$$

and let

$${{\tilde{B}}}_q(A,n)=\int _0^{\infty \cdot (1-q)}{\left( -\lambda xq^{A+nI};q\right) _\infty \over (-\lambda x;q)_\infty }x^{A-I}d_qx,\quad n\in {\mathbb{N}}$$

It easy to show that

56 $$\lim _{n\rightarrow \infty }{{\tilde{B}}}_q(A,n)=(1-q)^A{\tilde{\Gamma }}_q(A)$$

From the definition of *q*-derivative (3), we get

$$D_q\left\{ {\left( -\lambda xq^{A+nI};q\right) _\infty \over (-\lambda x;q)_\infty }x^{A+nI}\right\} =[A+nI]_q{\left( -\lambda xq^{A+(n+1)I};q\right) _\infty \over (-\lambda x;q)_\infty }x^{A+(n-1)I}$$

On *q*-integrating by parts with using the results obtained in Lemma 2 and the above result, we obtain recursive formula

$$\begin{aligned} {{\tilde{B}}}_q(A,n+1)&=[A+nI]_q^{-1}q^n\int _0^{\infty \cdot (1-q)}(qx)^{-n}D_q\left\{ {\left( -\lambda xq^{A+nI};q\right) _\infty \over (-\lambda x;q)_\infty }x^{A+nI}\right\} d_qx\\&=-q^n[A+nI]_q^{-1}[-n]_q\int _0^{\infty \cdot (1-q)}{\left( -\lambda xq^{A+nI};q\right) _\infty \over (-\lambda x;q)_\infty }x^{A-I}d_qx\\&=[n]_q[A+nI]_q^{-1}{{\tilde{B}}}_q(A,n),\quad n\in {\mathbb{N}}\end{aligned}$$

which can be read as

57 $${{\tilde{B}}}_q(A,n)=(q;q)_{n-1}\left( q^{A+I};q\right) _{n-1}^{-1}{{\tilde{B}}}_q(A,1),\quad n\in {\mathbb{N}}$$

Also the function ${{\tilde{B}}}_q(A,1)$ can be computed as

$$\begin{aligned} {{\tilde{B}}}_q(A,1)&=[A]_q^{-1}\int _0^{\infty \cdot (1-q)}D_q\left\{ {\left( -\lambda xq^{A};q\right) _\infty \over (-\lambda x;q)_\infty }x^{A}\right\} d_qx\\&=[A]_q^{-1}\left\{ {\left( -\lambda xq^{A};q\right) _\infty \over (-\lambda x;q)_\infty }x^{A}\right\} _0^{\infty \cdot (1-q)} \end{aligned}$$

Replacing $x$ by $(1-q)q^{-n}$ yields

$${{\tilde{B}}}_q(A,1)=[A]_q^{-1}(1-q)^A\lim _{n\rightarrow \infty }\left[ q^{-nA}{\left( -\lambda (1-q)q^{A-nI};q\right) _\infty \over \left( -\lambda (1-q)q^{-n};q\right) _\infty }\right]$$

Let the function

$$(1-q)^{A-I}K_q(A,\lambda )=\lim _{n\rightarrow \infty }\left[ q^{-nA}{\left( -\lambda (1-q)q^{A-nI};q\right) _\infty \over \left( -\lambda (1-q)q^{-n};q\right) _\infty }\right] ^{-1}$$

Using (29) followed by (23) when $n=k$, we can deduce that

$$K_q(A,\lambda )=(1-q)^{I-A}(-\lambda (1-q);q)_\infty \left( {-q\over \lambda (1-q)};q\right) _\infty e_q\left( -\lambda q^A\right) \left( {-q^{I-A}\over \lambda (1-q)};q\right) _\infty$$

which concludes that

58 $$K_q(A,\lambda ){{\tilde{B}}}_q(A,1)=[A]_q^{-1}(1-q)$$

In view of (56)–(58), we obtain

$$K_q(A){\tilde{\Gamma }}_q(A)=(q;q)_\infty \left( q^A;q\right) _\infty ^{-1}(1-q)^{I-A}$$

An important relation for the *q*-gamma matrix function was obtained by Salem (2012) as

$$\Gamma _q(A)=(q;q)_\infty \left( q^A;q\right) _\infty ^{-1}(1-q)^{I-A},\quad q^{-k}\not \in \sigma \left( q^A\right) ,k\in {\mathbb{N}}_0$$

which reveals that

$$K_q(A){\tilde{\Gamma }}_q(A)=\Gamma _q(A)$$

This completes the proof.

The results proved in this section can be summarized in the following theorem:

### **Theorem 17**

*Let*$$n\in {\mathbb{N}}_0,$$$$\lambda$$*be a complex number with*$${\mathfrak{R}}(\lambda )>0$$*and*$$A\in {\mathbb{C}}^{r\times r}$$*satisfying the conditions* (12) *and*$$q^{-k}\not \in \sigma (q^A)$$*for all*$$k\in {\mathbb{N}}_0,$$*then the q-Laguerre matrix polynomials sequence*$$\{L_n^{(A,\lambda )}(x;q)\}_{n\ge 0}$$*is an orthogonal matrix polynomials sequence with respect to the inner product*

59 $$\left\langle L_n^{(A,\lambda )}(x;q),L_m^{(A,\lambda )}(x;q)\right\rangle ={q^{\left( \begin{array}{c}n\\ 2\end{array}\right) }\lambda ^n\over [n]_q!}q^{nA}K_q^{-1}(A+(n+1)I,\lambda )\Gamma _q(A+(n+1)I)\delta _{mn}.$$

## Conclusion

In our work, we introduce the *q*-Laguerre matrix polynomials (19) hold for ${\tilde{\mu }}(A)>-1$ which verifies the second-order matrix difference equation (6). Four generating functions of this matrix polynomials are investigated. Two slightly different explicit forms are introduced. Three-term recurrence relation, Rodrigues-type formula and the *q*-orthogonality property are given.
